# Quo Vadis Botulinum Toxin: Normative Constraints and Quality of Life for Patients With Idiopathic OAB?

**DOI:** 10.3389/fsurg.2018.00061

**Published:** 2018-10-16

**Authors:** Sandra Mühlstädt, Shahidul Mischner, Jennifer Kranz, Petra Anheuser, Nasreldin Mohammed, Joachim A. Steffens, Paolo Fornara

**Affiliations:** ^1^Department of Urology and Kidney Transplantation, Martin-Luther-University, Halle (Saale), Germany; ^2^Department of Orthopaedics, Berufsgenossenschaftliche Kliniken Bergmannstrost, Halle (Saale), Germany; ^3^Department of Urology and Paediatric Urology, St. Antonius Hospital, Eschweiler, Germany; ^4^Department of Urology, Asklepios Clinic, Hamburg, Germany

**Keywords:** idiopathic OAB, urge incontinence, anticholinergics, botulinum toxin, quality of life

## Abstract

**Background:** Idiopathic overactive bladder (iOAB), with or without urge incontinence (UI), has significant psychosocial effects on patients' quality of life (QoL). The first choice of treatment for iOAB is anticholinergics and, alternatively, the β-3-adrenoceptor agonist mirabegron. However, systemic side effects and contraindications should be considered for both medications.

**Objective:** We report the efficacy, safety and effects on QoL of botulinum toxin therapy (onabotulinum toxin type A, BOTOX®, Allergan) among patients with iOAB ± UI.

**Patients and Methods:** Between 2005 and 2013, 51 patients were treated with onabotulinum toxin A (100 units). The inclusion criteria were the presence of confirmed iOAB ± UI with previous use of anticholinergic medication. Micturition frequency, pad count, postvoid residual volume and QoL were evaluated using two validated questionnaires [the Client Satisfaction Questionnaire-8 (CSQ-8) and the King's Health Questionnaire (KHQ)]. Statistical analysis was performed with SPSS 24.0 (*p* < 0.05).

**Results:** After botulinum toxin injection, a significant improvement in iOAB ± UI symptoms was observed. The micturition frequency decreased from 10.4 ± 0.5 to 5.2 ± 0.4 micturitions per day (*p* = 0.026), and the pad count decreased from 3.6 ± 1.0 to 1.2 ± 0.3 pads per day (*p* = 0.033). Anticholinergics were not used during the administration of botulinum toxin therapy. Complications and postoperative need for intermittent self-catheterization (ISC) were not observed. Overall, 72 and 24% of patients reported being “satisfied” or “very satisfied” with the treatment. Additionally, 66% of patients would choose botulinum toxin again for the treatment of iOAB.

**Conclusion:** Botulinum toxin therapy is an efficient, safe, and life-improving treatment for iOAB.

## Background

Idiopathic overactive bladder (iOAB), with or without consecutive urge incontinence (UI), has a high prevalence of ~12–19% (1, 2) and has significant psychosocial effects on the quality of life (QoL) of affected patients (3, 4). In addition to a change in lifestyle and physiotherapy, anticholinergics and, since 2014 in Germany, the β-3 adrenoceptor agonist mirabegron are the first-line therapeutic options. Although the (selective) efficacy of oral medication has improved in recent years, systemic side effects and contraindications must be considered. For intolerable side effects and contraindications or after failed conservative drug therapy, more invasive treatment options, such as transurethral injection of botulinum toxin (onabotulinum toxin type A, BOTOX®, Allergan), testing of sacral neuromodulation (peripheral nerve evaluation, two-stage testing, Interstim II®, Medtronic) and bladder augmentation or urinary diversion as a last resort, depending on the severity of the OAB symptoms, are available (5–7). In addition, recent studies have shown that anticholinergics can negatively affect cognitive processes, especially in elderly patients (8, 9). Considering the high prevalence of iOAB, the mentioned therapeutic options, and the observed side effects and contraindications of oral medication, including their sometimes exorbitant therapy costs, the economic aspects of the treatment of iOAB have also become increasingly important. Botulinum toxin seems to be well-suited for the treatment of iOAB. We report on the efficacy, safety and effects on QoL of onabotulinum toxin type A therapy among patients with iOAB ± UI.

## Patients and methods

The present study is a retrospective observational study. A total of 51 patients were evaluated. Between 2005 and 2013, the patients were treated with onabotulinum toxin type A for iOAB ± UI after failure of conservative treatment (lifestyle modification, pelvic floor exercises and anticholinergic medication). All patients received 100 units of onabotulinum toxin type A (BOTOX®, Allergan), disturbed on 20 intradetrusor injections, excluding the trigone and the bladder dome. The injections were performed with a rigid endoscope under general anesthesia. The surgical technique was the same in each patient. The inclusion criteria of the study included the presence of diagnostically confirmed iOAB ± UI with previous administration of conservative therapy. Patients with pre-existing neurological conditions or evidence of detrusor overactivity in the urodynamic study were excluded from the study. For each patient the first follow up took place 3 months after the botulinum toxin injection. The further follow up examinations were carried out every 12 months. Each follow up included a 3 days-voiding diary, with 24-h micturition frequency and 24-h pad count, as well as the postvoid residual volume, as recommended in the EAU guidelines (10). For the study these objective parameters were evaluated based on the electronic documentation. QoL and general patient satisfaction were analyzed by means of two validated questionnaires [the Client Satisfaction Questionnaire-8 (CSQ-8) and the King's Health Questionnaire (KHQ)], which were sent to the patient by post. The questionnaire response rate was 98%.

The CSQ-8 is a validated questionnaire that determines patient satisfaction in terms of treatment in general, treatment course and success, and the quality of treatment received. The CSQ-8 has been evaluated in several studies and shows good psychometric properties (11, 12). It consists of eight questions, each with four answer options (e.g., very satisfied, satisfied, moderate, not satisfied), which are represented by numerical values from one to four. The higher the total score is, the higher the patient satisfaction.

The KHQ is a specific and validated questionnaire that evaluates the QoL of patients with UI. It was designed to assess the impact of incontinence problems on the daily life of those affected and is ideally suited to evaluate new treatments for UI. The KHQ has been evaluated in several studies and shows good validity and reliability (13, 14). It contains 32 questions, which are divided into three subgroups of questions. The first part refers to general health status and incontinence burden. The second part assesses the restrictions associated with UI in specific areas of daily life. The third part of the questionnaire evaluates the UI symptom burden and consists exclusively of questions on actual problems. A total KHQ score can be calculated, and this score is directly related to the QoL of the respondents. This score ranges from 0 (best) to 100 (very bad). The lower the score is, the higher the QoL and vice versa. The statistical analysis was performed with SPSS 24.0 for Windows, and the analysis of significance was performed using Student's *t*-test (*p* < 0.05). The data are presented as the mean, standard deviation and range.

## Results

The baseline characteristics of the population are shown in Table 1. The average age of the overall population was 63.5 ± 13.9 years (21–88 years), with patients over 60 years (*n* = 32) composing the largest group. Of the 51 patients, women represented the majority (*n* = 39). Male patients were significantly underrepresented at all ages. The mean body mass index (BMI) of the total population was 27.1 ± 3.6 kg/m^2^ (20–36 kg/m^2^); the highest BMI values were ~27.9 ± 3.7 kg/m^2^ and were found in the cohort of patients older than 60 years.

**Table 1:** Baseline characteristics of the patient population.

**Patient age (years)**	**Women (n)**	**Men (n)**	**BMI (kg/m^2^)**
21–30	2	0	23.5 ± 2.5
31–40	1	1	27 ± 2
41–50	3	1	22 ± 2.2
51–60	8	3	27 ± 2.2
>60	25	7	27.9 ± 3.7
Total	39	12	27.1 ± 3.6

Prior to starting transurethral botulinum toxin injection, all 51 patients first received conservative therapy with lifestyle intervention (drinking protocol, voiding and dietary recommendations), physiotherapy and anticholinergics. Of the patient population, 51% received fesoterodine fumarate (Toviaz®); 29%, propiverine hydrochloride (Mictonorm®); and 20%, oxybutynin hydrochloride (Oxybutunin®). Overall, 70% of patients took anticholinergic medication for 12 months or less.

**Figure 1:**
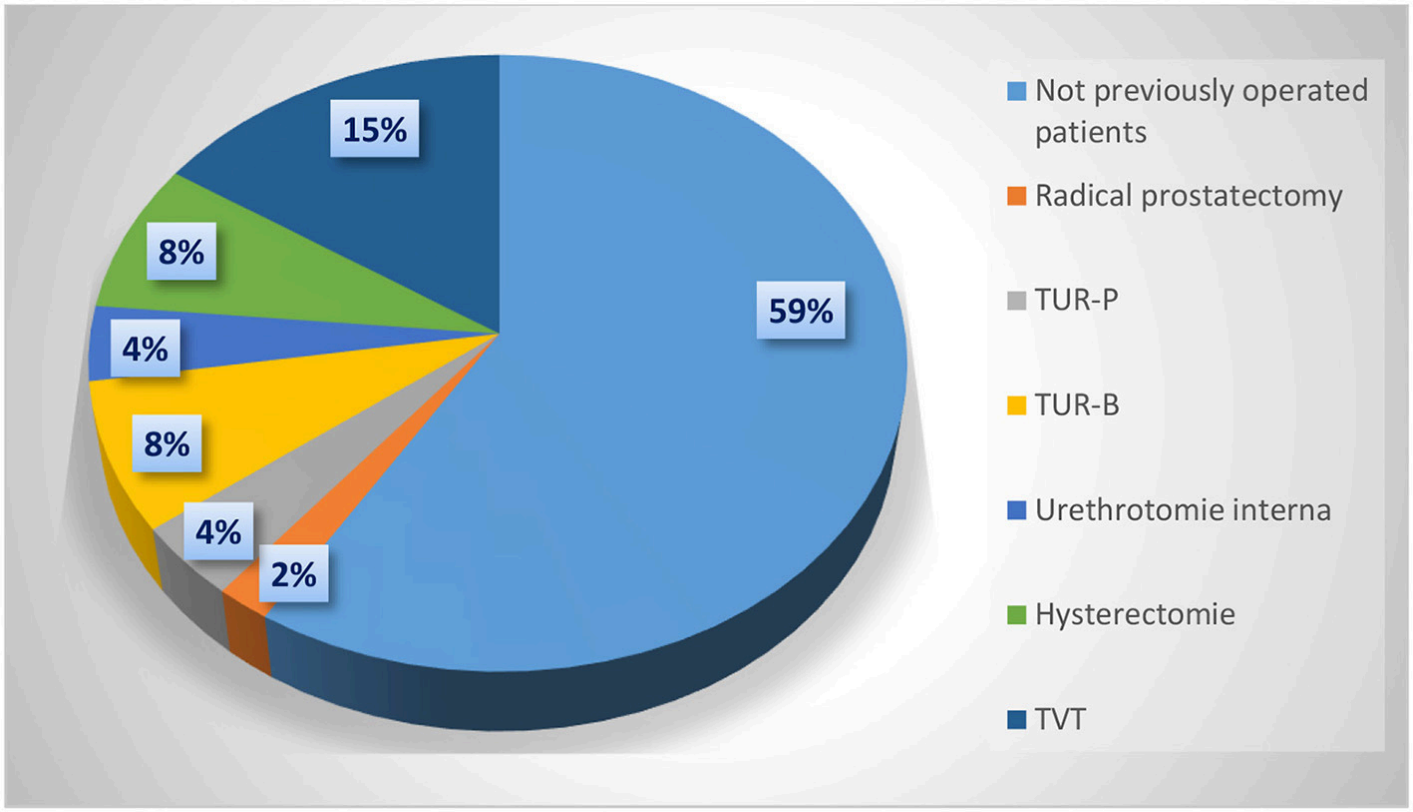
Previous urogynecological surgery.

Patients who had not received prior urogynecological surgery composed the largest group of patients. In total, 21 patients (41%) had undergone previous urogynecological surgery, including 15 women and six men. The distribution of previous operations is shown in Figure 1. The extent to which previous operations represented a risk factor for the development of iOAB could not be clearly derived from the presented data.

The perioperative parameters of the population are shown in Table 2. Regarding the operation time, the mean duration of transurethral botulinum toxin injection was 17.1 ± 1.9 min (14–21 min) for the total population. Overall, 51% of the patients (26/51) received one repeated botulinum toxin injection; 27% of these patients (7/26) had a second repetition of the procedure; finally, only three of these seven patients had a third repeated procedure. The average intervals between the individual botulinum toxin injections were 12 months. As expected, no intraoperative complications were observed. Furthermore, no patients developed any postoperative complications, and no patients needed postoperative intermittent self-catheterization (ISC).

**Table 2:** Perioperative parameters of the patient population.

	**1st BOTOX^®^-injection**	**2nd BOTOX^®^-injection**	**3rd BOTOX^®^-injection**	**4th BOTOX^®^-injection**
Number of patients (n)	51	26	7	3
Proportion of women (n)	39	19	7	3
Proportion of men (n)	12	7	0	0
Operative time (min)	17.1 ± 1.9	16.4 ± 1.7	16.6 ± 1.4	16.5 ± 1.1
Injection interval (months)	–	10.6 ± 1.8	12.3 ± 0.2	11.7 ± 0.9

**Figure 2:**
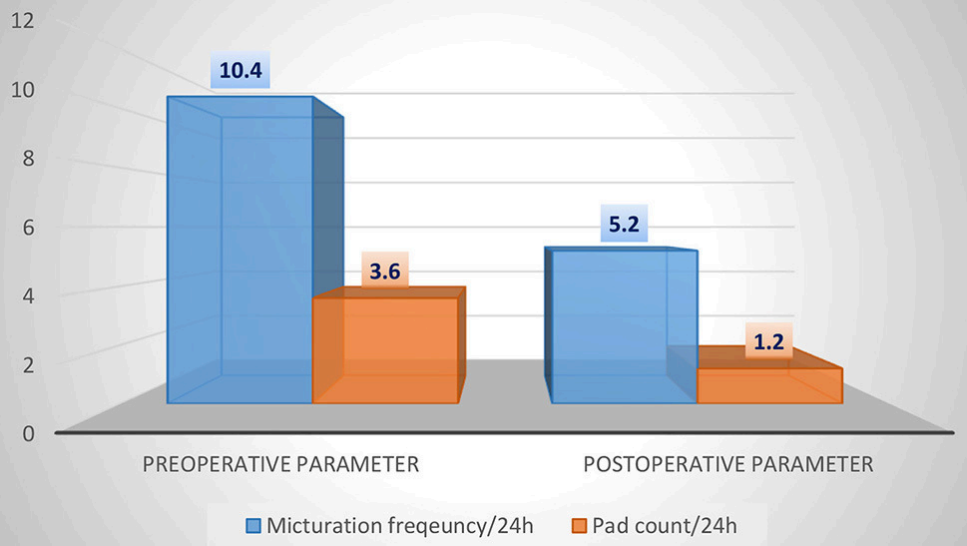
Comparison of pre- and postoperative 24-h micturition frequency and 24-h pad count.

The 24-h micturition frequency decreased by ~50% in the pre/postoperative comparison. The mean frequency of micturitions postoperatively was 5.2 ± 0.4 micturitions per 24 h, whereas preoperatively, 10.4 ± 0.5 micturitions per 24 h were observed (*p* = 0.026). Similarly, for the pad count, we found a significant reduction of ~67% in the pre/postoperative comparison. The mean pad count was only 1.2 ± 0.3 (0–2) pads per 24 h postoperatively, whereas 3.6 ± 1.0 (1–6) pads per 24 h were used preoperatively (*p* = 0.033). In summary, as shown in Figure 2 and Table 3, we found a significant reduction in the frequency of 24-h micturition as well as UI episodes. Despite the muscle-relaxing effect of the botulinum toxin, we found no significant change in the postvoid residual volume in the pre/postoperative comparison. In the overall population, the mean pre- and postoperative postvoid residual volume was ~50.7 ± 1.6 ml (20–90 ml) and 48.3 ± 6.3 ml (0–70 ml), respectively (*p* = 1.0998).

**Table 3:** Comparison of pre- and postoperative 24-h micturition frequency, 24-h pad count and residual voiding volume (*student's *t*-test, α = 0.05).

	**Preoperative parameters**	**Postoperative parameters**	p-Wert*
Micturition frequency/24 h	10.4 ± 0.5	5.2 ± 0.4	0.026
Pad count/24 h	3.6 ± 1.0	1.2 ± 0.3	0.033
Residual voiding volume (ml)	50.7 ± 1.6	48.3 ± 6.3	1.0998

**Figure 3:**
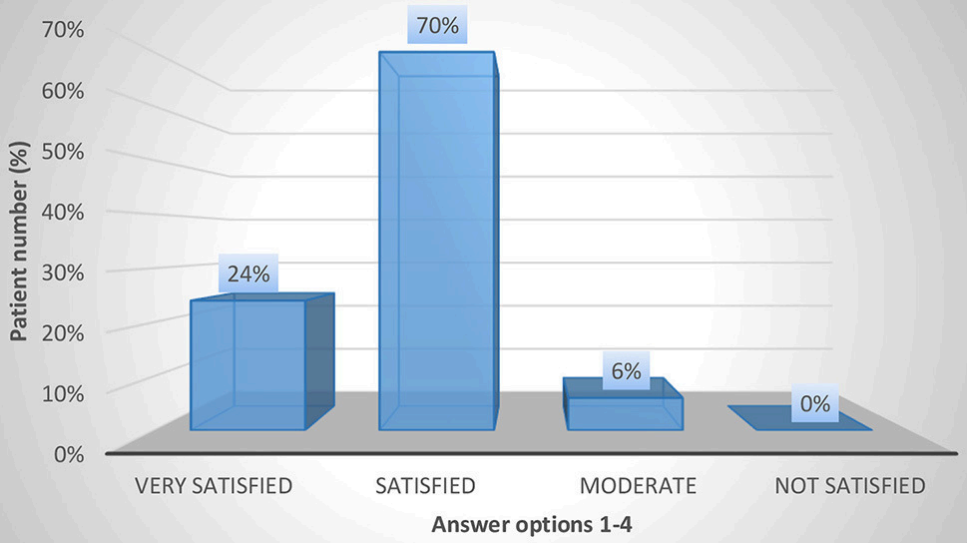
CSQ-8 question 5: How satisfied were you with the course of treatment?.

**Figure 4:**
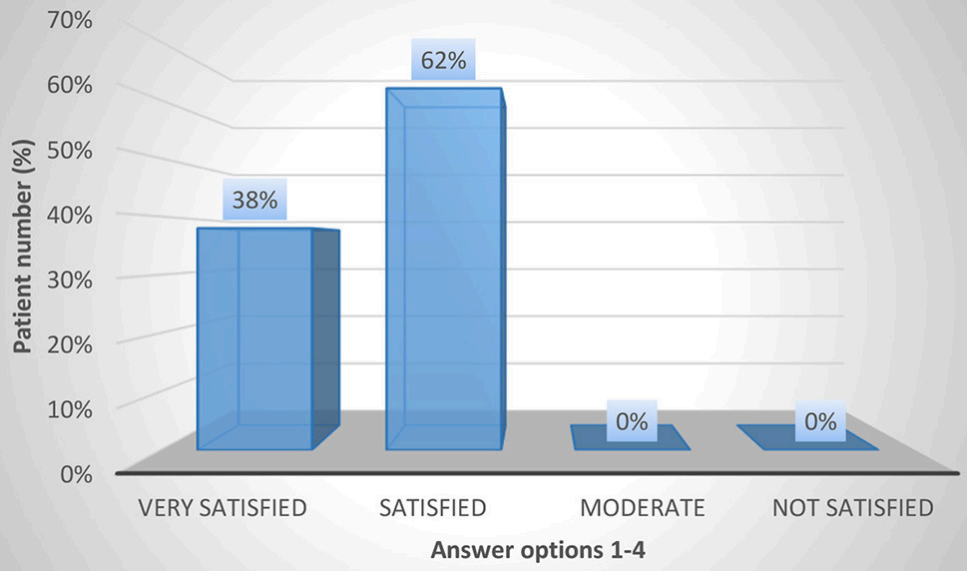
CSQ-8 question 6: How satisfied were you with the overall therapy and the result of the therapy?.

**Figure 5:**
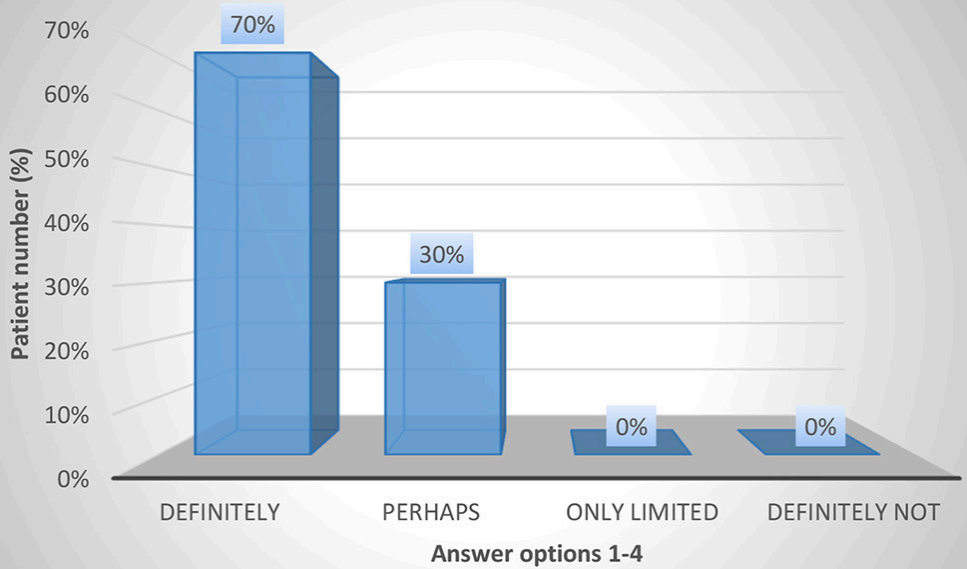
CSQ-8 question 8: Would you choose this type of treatment again?.

The results regarding patient satisfaction and QoL using the CSQ-8 and KHQ are shown in Figures 3–5 and in Tables 4, 5. In the overall population, 24 and 72% of the patients stated that they were “very satisfied” or “satisfied,” respectively, with the course of the botulinum toxin treatment (CSQ-8 question 5). Furthermore, 100% of the patients stated that they were “very satisfied” or “satisfied” with the received therapy (CSQ-8 question 6) and that they would “definitely” or “perhaps” decide to receive the treatment again (CSQ-8 question 8); of these, 66% of patients responded with “definitely” and 34% with “perhaps.” However, the procedure was repeated in only 51% of the patients. Regarding the KHQ results, question complexes K3 to K6 were particularly representative. The third question complex concerns the limitations in daily activities due to the disease (K3). It addresses the extent of harm both within and outside of the home. For the overall population, the mean K3 score was only 15.3 (0–33). Thus, the limitations in everyday activities during botulinum toxin therapy are low. Physical and social limitations are addressed in question complexes K4 and K5. For the overall group, the scores for K4 and K5 were 13.3 (0–33) and 8.9 (0–11), respectively. Thus, in comparison to question complex three, which generally assesses limitations in daily activities, the physical and social limitations under botulinum toxin therapy are even lower. Question complex K6, which addresses personal relations, also showed a very low mean value of 11.9 (0–12). In summary, the symptoms of iOAB ± UI appear to be significantly reduced under botulinum toxin therapy.

**Table 4:** Evaluation of general patient satisfaction using the CSQ-8 questionnaire.

**Question**	**Answer**			
	**4**	**3**	**2**	**1**
1. How would you rate the quality of the treatment you received?	Excellent **13/50 (26%)**	Good **37/50 (74%)**	Low 0	Very low 0
2. Did you get the kind of treatment you wanted?	Definitely **33/50 (66%)**	Yes, generally **17/50 (34%)**	No, not really 0	Definitely not 0
3. To what extent has the treatment met your expectations?	Almost all **14/50 (28%)**	Most of **36/50 (72%)**	Only a few 0	None 0
4. Has the treatment helped you to cope better with your disease?	Excellent **16/50 (32%)**	Good **30/50 (60%)**	Low **4/50 (8%)**	Very low 0
5. How satisfied were you with the course of treatment?	Very satisfied **12/50 (24%)**	Satisfied **35/50 (70%)**	Moderate **3/50 (6%)**	Not satisfied 0
6. How satisfied were you with the overall therapy and the result of this?	Very satisfied **19/50 (38%)**	Satisfied **31/50 (62%)**	Moderate 0	Not satisfied 0
7. If a friend of yours has the same problem, would you recommend the treatment?	Definitely **33/50 (66%)**	Perhaps **17/50 (34%)**	Only limited 0	Definitely not 0
8. Would you choose this type of treatment again?	Definitely **35/50 (70%)**	Perhaps **15/50 (30%)**	Only limited 0	Definitely not 0

*Bold values the results were highlighted*.

**Table 5:** Evaluation of QoL by means of the KHQ questionnaire.

**Topics**	**Number of questions**	**Score (mean ± standard deviation)**	**Range**
General health perceptions (K1)	1	21.5 ± 8.7	0–25
Incontinence impact (K2)	1	21.3 ± 15.9	0–33
Limitations in daily life (K3)	2	15.3 ± 9.9	0–33
Physical limitations (K4)	2	13.3 ± 12.1	0–33
Social limitations (K5)	3	8.9 ± 4.4	0–11
Personal relationships (K6)	2	11.9 ± 7.5	0–12
Emotions (K7)	3	7.8 ± 5.6	0–22
Sleep/energy (K8)	2	13.3 ± 7.5	0–33
Severity measures (K9)	4	21.5 ± 9.7	0–33

## Discussion

Before recognizing the therapeutic benefits of botulinum toxin, it was considered only a highly potent poison resulting from inadequate food preservation that led to serious neurological symptoms, called botulism (15). Only one gram of botulinum toxin can kill one million people (16). Despite its destructive potential as a poison, botulinum toxin has a broad spectrum of applications in current medicine and is used for several diseases. Botulinum toxin was first applied in this way by the physician AB Scott for the treatment of strabismus (17). In the past two decades, the field of urology has also discovered the benefits of botulinum toxin. Its efficacy for neurogenic overactive bladder (nOAB) was first recorded in 2000 (18). Other international studies have confirmed its efficacy for the treatment of neurogenic bladder dysfunction, with the Food and Drug Administration (FDA) confirming the use of botulinum toxin in therapy-refractory nOAB in 2011 (19). However, what began as a miracle weapon for the treatment of neurogenic detrusor hyperactivity quickly became a nerve-racking ordeal for patients and practitioners in Germany when it came to being reimbursed for its use for iOAB, despite receiving a safety evaluation and approval by the Bundesinstitut für Arzneimittel und Medizinprodukte (BfArM) in 2013. These challenges most likely occurred because the use of botulinum toxin for iOAB was equated to the “esthetic benefit” of receiving plastic surgery. Thus, iOAB, whether wanted or not, was ultimately equated to a “lifestyle phenomenon.” This occurred in a treatment era in which we attach great importance to the term “quality of life” of our patients. However, what is QoL except a now “overstretched” term? According to the World Health Organization (WHO), QoL is “the subjective perception of a person about their position in life, their relation to the culture and value systems in which the person lives, and their goals, expectations, standards and concerns.” In slightly more simplified terms, we also tend to refer to QoL as “subjective well-being.” However, from the general scientific perspective, it is considered a multidimensional construct that can be depicted by its subdomains by means of indicators and specific questions. The presentation of these subdomains, also in medicine, is increasingly difficult but essential because it generates valuable information about patients' feelings about their disease or therapy.

Various studies have shown that the QoL of patients with iOAB is significantly reduced (3, 4, 20) when compared with that of the general population. Lia et al. (4) found that 27.5% of iOAB patients interviewed had depression. In comparison, the lifetime prevalence of depression is generally only 15%. This discrepancy implies a significant correlation between OAB and depression and between OAB and QoL.

For the treatment of iOAB itself, a change in lifestyle, physiotherapy and the use of anticholinergics or mirabegron are the first-line treatment options. Cognitive deficits (8, 9) and contraindications, such as myasthenia gravis and cardiovascular diseases should be noted for both groups of drugs, including systemic adverse reactions, such as dry mouth, constipation, and cardiac arrhythmia for those taking anticholinergics. In the case of intolerable side effects, contraindications or a failure of conservative therapy, more invasive treatment options depending on the severity of the iOAB symptoms are available, such as transurethral injection of botulinum toxin (onabotulinum toxin type A, BOTOX®, Allergan) and the testing of sacral neuromodulation (peripheral nerve evaluation, two-stage testing, Interstim II®, Medtronic) up to bladder augmentation or urinary diversion (5–7). However, the latter therapy represents the ultima ratio. Considering the high prevalence of iOAB, the systemic side effects and the contraindications of oral medication, including their sometimes exorbitant treatment costs, the economic aspects of iOAB treatment have become increasingly important. Botulinum toxin seems to be particularly well-suited for the treatment of iOAB. In the present study, we evaluated 51 patients treated with botulinum toxin (100 units onabotulinum toxin type A) for conservative drug-refractory iOAB ± UI.

We found that transurethral botulinum toxin injection significantly reduced the frequency of micturition and the use of incontinence pads. The mean frequency of micturition decreased from 10.4 ± 0.5 preoperatively to 5.2 ± 0.4 micturitions per 24 h postoperatively (*p* = 0.026). In concordance, the mean pad count decreased from 3.6 ± 1.0 to 1.2 ± 0.3 pads per 24 h (*p* = 0.033). There was also a significant improvement in QoL and overall patient satisfaction. Limitations in daily activities, physical and social limitations and limitations in personal relationships showed a very favorable value constellation for therapy with botulinum toxin. Additionally, all patients stated being “satisfied” or “very satisfied” with the received therapy, and 66% of the patients would “definitely” opt to receive botulinum toxin injection again. Similar results were found in studies by Fowler et al. (21), Chapple et al. (22), and Malde et al. (12). The study by Fowler et al. (21) included 313 patients and randomized different onabotulinum toxin doses with placebo. That study showed a significant improvement in health-related as well as UI-specific QoL [according to the KHQ and incontinence QoL (I-QOL)] for onabotulinum toxin compared to placebo. The multi-center, double-blinded and randomized study by Chapple et al. (22) also showed a significant reduction in the frequency of micturition and pad count and an equally significant improvement in health-related and UI-specific QoL. Furthermore, Malde and colleagues (12) found a similar result to our study (using the CSQ-8) in a single-center study. In addition, Miotla et al. (23) found an improvement in female sexuality for botulinum toxin therapy using the female sexual function index (FSFI) questionnaire. Of 56 patients, more than 90% reported improvement in all six domains of the FSFI.

Intra- and postoperative complications were not documented in our patient population. Additionally, no patients showed postoperative need for ISC. This is consistent with the data in the literature (24, 25). Miotla et al. (25) described a higher likelihood of postvoid residual volume of more than 200 ml or a need for ISC, but only among elderly patients with a higher rate of pregnancies and vaginal deliveries.

In summary, botulinum toxin improves the treatment of iOAB ± UI. However, the use of botulinum toxin in iOAB has been extremely difficult in daily clinical practice in Germany (26, 27); this difficulty may also explain why only 51% of the patients in our population received a repeat botulinum toxin injection. The introduction of the corresponding EBM accounting in Germany in January 2018 should hopefully provide a true remedy for this so that patients can now receive a botulinum toxin injection when indicated. In brief, the QoL of the patient and economic aspects should not be opposing ideas.

### Limitations of our study

Our study was limited by the retrospective nature and the lack of a control group. In addition, preoperative QoL was not analyzed in comparisons. Despite these limitations, our study shows that botulinum toxin is an effective treatment option for iOAB and should not be withheld from the patient.

## Conclusion

iOAB ± UI has a high disease prevalence of ~12–19%. Basic therapy for iOAB ± UI involves a change in lifestyle and physiotherapy as well as anticholinergics or mirabegron. In the presence of systemic side effects, contraindications and/or a failure of conservative (drug) therapy, more invasive treatment options may be available depending on the severity of OAB symptoms. In this study, onabotulinum toxin type A was shown to represent a tremendously effective, safe and extremely QoL-improving treatment option for iOAB ± UI.

## Ethics statement

This study was carried out in accordance with the recommendations of the Ethikkommission of the Martin-Luther University Halle-Wittenberg, with written informed consent from all subjects. All subjects gave written informed consent in accordance with the Declaration of Helsinki. The protocol was approved by the Ethikkommission of the Martin-Luther University Halle-Wittenberg.

## Author contributions

SaM: protocol development, data collection and analysis, manuscript writing. ShM: data collection and analysis, manuscript editing. JK, PA, NM, and JS: manuscript editing. PF: protocol development, manuscript editing.

### Conflict of interest statement

The authors declare that the research was conducted in the absence of any commercial or financial relationships that could be construed as a potential conflict of interest.
